# Metformin Administration Protects Against Deltoid Tendon Damage Through Activation of Notch Signaling

**DOI:** 10.1002/imt2.70074

**Published:** 2025-08-31

**Authors:** Can Liu, Runqi Wang, Yong Xu, Qingyi Liu, Yin Li, Xiangpeng Liu, Hui Shu, Kaige Gao, Xingye Zheng, Yingying Dong, Yijun Wang, Bin Guo, Lei Fu, Bin Zhang, Liang Zhao, Zhihao Jia, Xiaobo Sun

**Affiliations:** ^1^ Department of Shoulder and Elbow Surgery, Center for Orthopedic Surgery The Third Affiliated Hospital of Southern Medical University Guangzhou China; ^2^ Shoulder Research Institute, Academy of Orthopedics Guangzhou China; ^3^ Cambridge‐Suda Genomic Resource Center, Suzhou Medical College Soochow University Suzhou China; ^4^ Orthopedic Institute, Suzhou Medical College Soochow University Suzhou China; ^5^ Wisdom Lake Academy of Pharmacy Xi'an Jiaotong‐Liverpool University Suzhou China; ^6^ Institute of Medicinal Plant Development, Peking Union Medical College and Chinese Academy of Medical Sciences Beijing China; ^7^ Collaborative Innovation Center of Prevention and Treatment of Major Diseases by Chinese and Western Medicine Henan Province China; ^8^ Academy of Chinese Medical Science Henan University of Chinese Medicine Zhengzhou China; ^9^ The First Affiliated Hospital of Henan University of Chinese Medicine Zhengzhou China

## Abstract

Type 2 Diabetes Mellitus (T2DM) is a growing global health concern that is associated with severe complications including diabetic tendinopathy. In this study, we found that T2DM patients had a significantly higher prevalence of tendon surgery compared to non‐T2DM patients, which were alongside impaired ECM and cell adhesion. Notably, metformin‐treated T2DM patients had a lower prevalence of tendon surgery compared to other medications, along with improved tendon fiber structure, downregulation of tendon damage marker *MMP3*, and upregulation of *HES1*, a Notch signaling effector gene. Metformin also activates Notch signaling in cultured tenocytes, and tendons from diabetic mice and aged monkey. These findings highlight metformin's potential to protect tendons by activating Notch signaling, offering novel insights into its therapeutic benefits beyond glucose regulation.

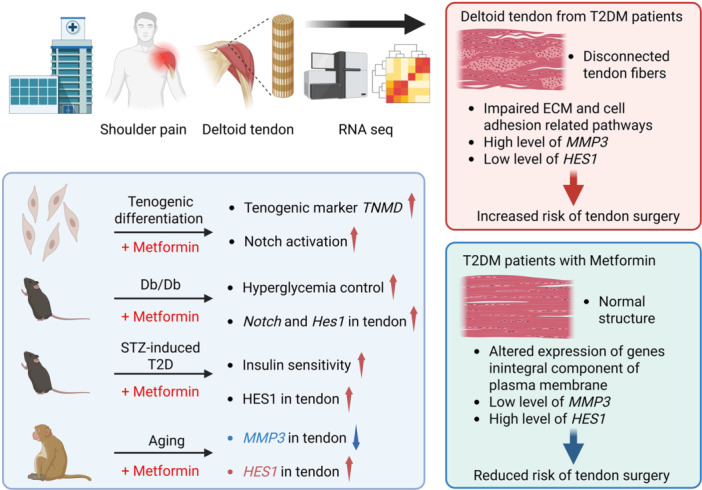

To the Editor,

The global prevalence of type 2 diabetes mellitus (T2DM) is rising rapidly, contributing to significant mortality from complications, including chronic kidney disease, cardiovascular disease, heart failure, and diabetic tendinopathy [1]. Tendons are dense, organized fibrous connective tissues primarily composed of type I collagen. They connect muscles to bones and enable efficient force transmission during movement. According to a high‐resolution ultrasound‐based study, 84.6% of T2DM patients have disorganized fibers in their Achilles tendon after over 10 years of diagnosis [2]. Epidemiological studies consistently show a higher incidence of tendinopathy in diabetic patients compared to age‐ and sex‐matched nondiabetic controls [3]. Tendinopathy is a major cause of chronic pain, limited joint range of motion, and tendon rupture in advanced cases among individuals with T2DM [4]. Despite extensive research into the structure, composition, imaging features, biomechanical properties, and histopathological changes of tendons in clinical and animal models of diabetes, effective management of diabetic tendinopathy remains limited [5]. This shortfall is primarily attributed to an incomplete understanding of the pathological and molecular mechanisms driving diabetic tendinopathy progression [6]. These findings underscore the urgent need to elucidate the molecular and cellular mechanisms underlying diabetic tendinopathy to enable the development of targeted and effective therapies.

Metformin is the first‐line treatment for T2DM in most clinical guidelines; however, its precise molecular mechanisms remain incompletely understood. Recent research has expanded metformin's potential applications beyond glycemic control to weight management, polycystic ovary syndrome, cancer prevention, neurodegenerative diseases, and antiaging [7, 8]. Animal studies have demonstrated that metformin administration mitigates tendinopathy caused by mechanical overloading and delays aging‐associated tendon degeneration [9, 10]. Additionally, a meta‐analysis revealed an association between metformin use and a reduced risk of rotator cuff disease in T2DM patients [11]. These findings suggest that metformin may play a protective role in diabetic tendinopathy. However, whether this effect arises from direct action on tenocytes or indirectly through improved glycemic control and insulin sensitivity remains unclear. In this study, we use RNA‐seq of human samples alongside cell and animal models to investigate the mechanisms by which metformin exerts beneficial effects on tendons.

### Patients with type 2 diabetes undergo deltoid tendon damage

We first collected outpatient data from the Department of Shoulder and Elbow Surgery, Center for Orthopedic Surgery, The Third Affiliated Hospital of Southern Medical University between 2021 and 2023. In total, 606 out of 4481 patients (13.5%) underwent surgery to repair tendon damage (Figure 1A,B), in which individuals with T2DM had a higher prevalence of tendon surgery (67 out of 245, 27.3%) (Figure 1A,B). We then collected uninjured tendon samples from the tendinous bands inserting into the acromion during deltoid tendon surgery (Figure S1A). H&E staining revealed that tendon fibers were tightly connected to elongated cells in healthy individuals, whereas in T2DM patients, tendon fibers were disconnected (Figure 1C).

**FIGURE 1:**
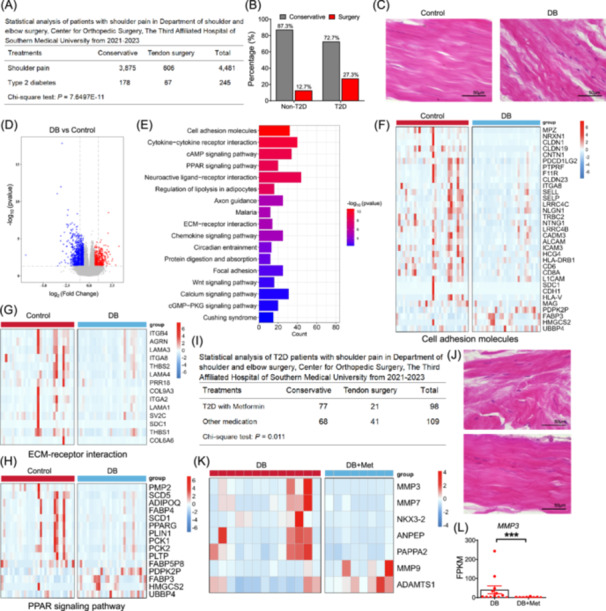
Patients with type 2 diabetes undergo deltoid tendon damage. (A) Statistical analysis of patients with shoulder pain. (B) Percentage of non‐type 2 diabetes mellitus (T2DM) and T2DM patients with conservative treatment or undergo tendon surgery. (C) Relative image of H&E staining using deltoid tendons from non‐T2DM and T2DM patients. (D) Volcano plot of the RNA‐seq results from Control and DB groups, *n* = 32 and 30, respectively. (E) KEGG analysis of deltoid tendon differentially expressed genes (DEGs). Heatmap of DEGs from Control versus DB that enriched in cell adhesion molecules (F), ECM–receptor interaction (G), and PPAR signaling (H) pathways. (I) Statistical analysis of T2DM patients with shoulder pain taking metformin or other medications. (J) Relative image of H&E staining using deltoid tendons from T2DM patients and T2DM patients with metformin. (K) Heatmap of the expression of genes in ECM–receptor interaction pathway. (L) Relative mRNA levels of *MMP3* from DB and DB+Met groups, *n* = 13 and 8, respectively. Data represent mean ± SEM (*t*‐test: ***p < 0.001).

We then collected 77 deltoid tendon samples for RNA sequencing. The samples were divided into four groups: Control, overweight‐nondiabetic (OW), obesity‐nondiabetic (OB), and Diabetes (DB), based on their BMI and diabetes diagnosis (Figure S1B). In total, we identified 2051 differentially expressed genes (DEGs) between the Control and DB groups, 806 DEGs between Control and OB groups, 884 DEGs between Control and OW groups, and 1181 DEGs between OW and OB groups (Figure S2) (Tables S1–S5). Gene ontology (GO) analysis revealed that genes involved in the extracellular space were significantly altered (Figure S3A–D). A total of 50 DEGs were overlapped between DB and OB groups compared to the Control group, respectively (Figure S4A). KEGG functional analysis revealed that most of these DEGs were enriched in immune‐related pathways, including viral protein interaction with cytokines and cytokine receptors, cytokine‐cytokine receptor interaction, and chemokine signaling pathways (Figure S4B). GO analysis also revealed that the shared DEGs were mainly enriched in neutrophil chemotaxis and extracellular space pathways (Figure S4C). The heatmap showed that the expression levels of several chemokines, such as *CCL24*, *CCL3*, *CCL4*, and *CXCL11*, were significantly reduced in the deltoid tendons of OB and DB individuals compared to the Control group (Figure S4D).

Next, we focused on the gene expression changes in the deltoid tendons of T2DM patients (Figure 1D). KEGG functional analysis revealed that the DEGs were most significantly enriched in cell adhesion molecules (Figure 1E). Heatmap showed that most of the DEGs related to cell adhesion molecules were downregulated (Figure 1F). Similarly, DEGs in ECM–receptor interaction pathway also showed a downregulation pattern (Figure 1G). Significant changes of genes in PPAR signaling pathway were also observed, including key genes involved in adipogenic differentiation, lipid synthesis and gluconeogenesis (Figure 1H). In addition, we also re‐analyzed the data using only female individuals and obtained similar pathway change (Figure S5A–D). These results demonstrate that diabetes causes gene expression change related to cell adhesion and extracellular matrix.

### Metformin administration ameliorates deltoid tendon damage and activates Notch signaling

We found that 21 out of 98 T2DM patients (21.4%) taking metformin required tendon surgery, which was significantly lower than the rate in T2DM patients on other medications (41 out of 109, 37.6%) (Figure 1I). H&E staining showed that the disrupted tendon fiber structure in diabetic deltoid tendons was ameliorated by metformin administration (Figure 1J). We then compared T2DM patients with (DB+Met) or without (DB) metformin administration (Figure S6A) and identified 878 DEGs (Figure S6B, Table S6). KEGG analysis revealed that these DEGs were enriched in ECM‐receptor interaction and immune‐related pathways (Figure S6C). GO analysis revealed that these DEGs were most significantly enriched in the “Integral component of plasma membrane” and “Extracellular region” pathways (Figure S7). We found that *MMP3* and *MMP7* decreased, while *MMP9* and *ADAMTS1* increased after metformin administration in T2DM patients (Figure 1K,L). Similarly, the expression level of *MMP3* was significantly reduced in tendon of aged monkeys with metformin (Figure S8A).

We observed that the most significant DEG between the DB+Met and DB group was *HES1* (Figure 2A), a key gene in Notch signaling. Western blot analysis confirmed that HES1 protein was higher in T2DM patients receiving metformin (Figure 2B). The expression level of HES1 was significantly increased in the supraspinatus tendons of aged monkeys administered metformin (Figure S8B,C).

**FIGURE 2:**
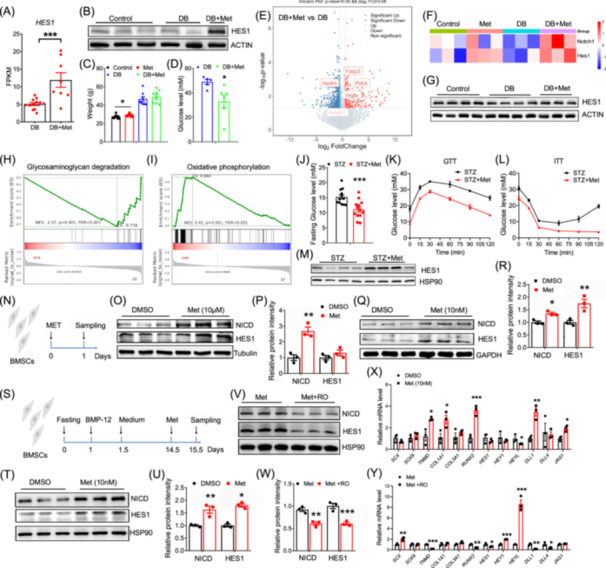
Metformin administration ameliorates deltoid tendon damage and activates Notch signaling. (A) Relative mRNA levels of *HES1* expression in type 2 diabetes mellitus (T2DM) patients (*n* = 13) versus Met‐treated T2DM patients (*n* = 8). (B) HES1 protein levels in deltoid tendons of Control, T2DM, and Met‐treated T2DM groups. (C) Body weight of WT and DB/DB mice after 3‐month of metformin treatment from WT and DB/DB groups. (D) Serum glucose levels of DB/DB mice with or without metformin, *n* = 5 and 6, respectively. (E) Volcano plot analysis of tendon transcriptomes in DB/DB mice versus Met‐treated DB/DB mice, *n* = 4. (F) Heatmap of Notch1 and Hes1 expressions in mouse deltoid tendon from WT, WT+Met, DB and DB+Met groups, *n* = 4. (G) Western blot analysis of HES1 protein levels in deltoid tendon from WT, DB and DB+Met groups. (H, I) Gene set enrichment analysis (GSEA) of DEGs from Control and Met‐treated DB/DB mice. (J) Fasting blood glucose in streptozotocin (STZ)‐induced T2DM mice, *n* = 13. (K) Glucose tolerance tests (GTT) in STZ‐T2DM mice, *n* = 5. (L) Insulin tolerance tests (ITT) in STZ‐T2DM mice, *n* = 5. (M) HES1 protein levels in tendon of Control and STZ‐induced T2DM mice, *n* = 4. (N) Metformin intervened bone marrow stem cells (BMSCs) were collected 24 h post‐intervention. Western‐blot analysis (O) of NICD and HES1 and quantification (P) after 10 μM metformin treatment, *n* = 3. Western‐blot analysis (Q) of NICD and HES1 and quantification (R) after 10 nM metformin treatment, *n* = 3. (S) A scheme showing metformin treatment on BMSCs after induction of tenogenic differentiation. Differentiated BMSCs were collected after sequential treatment: 24‐h nutrient deprivation, followed by 12‐h BMP‐12 exposure, culture in normal media until Day 14.5, 24‐h Met administration, and final sample collection. Western‐blot analysis (T) of NICD and HES1 and quantification (U) after 10 nM metformin treatment in tenogenic differentiated BMSCs, *n* = 3. Western‐blot analysis (V) of NICD and HES1 and quantification (W) after 10 nM metformin treatment in tenogenic differentiated BMSCs with or without notch inhibitor RO4929097, *n* = 3. (X) Relative mRNA expressions of *SCX*, *SOX9*, *TNMD*, *COL1A1*, *COL3A1*, *RUNX2*, *HES1*, *HEY1*, *HEYL*, *DLL1*, *DLL4*, *JAG1* in BMSCs from DMSO or Met groups, *n* = 3. (Y) Relative mRNA expressions of *SCX*, *SOX9*, *TNMD*, *COL1A1*, *COL3A1*, *RUNX2*, *HES1*, *HEY1*, *HEYL*, *DLL1*, *DLL4*, *JAG1* in BMSCs from Met or RO4929097 groups, *n* = 3. Data represent mean ± SEM (*t*‐test, two‐way ANOVA, and ANCOVA: *p < 0.05, **p < 0.01, ***p < 0.001).

We validated the data using the DB/DB mouse model with metformin treatment. Metformin treatment resulted in a slight increase in body weight of control mice, while no change was observed in the DB/DB mice (Figure 2C). Cardiac ultrasonography revealed that metformin reduced the left ventricular internal diameter (LVID) at end‐diastole, with no effect on LVID at end‐systole (Figure S9A). Metformin treatment significantly reduced both day and night VO_2_ of DB/DB mice (Figure S9B,C). After treatment, we observed a significant reduction in food intake and increased urine pH of DB/DB mice (Figure S9D,E). Blood biochemistry analysis revealed that metformin treatment lowered serum glucose levels in DB/DB mice (Figure 2D), while serum lipids remained unchanged (Figure S9F,G).

RNA‐seq of deltoid tendon revealed that Notch1 was significantly upregulated after metformin treatment (Figure 2E) (Tables S7–S10). GO analysis of the DEGs revealed a significant change in genes involved in extracellular space of deltoid tendons after metformin treatment (Figure S10). Heatmap showed that *Notch1* and *Hes1* were increased after metformin treatment (Figure 2F). HES1 protein level was lower in the deltoid tendons of DB/DB mice compared to controls but increased after metformin treatment (Figure 2G). GSEA showed that DEGs involved in oxidative phosphorylation were upregulated, while those involved in glycosaminoglycan degradation were downregulated (Figure 2H,I). A STZ‐induced T2DM mouse model further confirmed the result that metformin treatment upregulated HES1 in tendon of T2DM mice (Figure 2J–M).

We next performed in vitro investigation using bone marrow stem cells (BMSCs), which retain tenogenic capacity (Figure 2N). We found that both low‐dose (10 nM) and high‐dose (10 μM) metformin treatment increased the protein levels of NICD (Figure 2O–R). Low dose of metformin upregulated HES1 (Figure 2Q,R). We then induced tenogenic differentiation of BMSCs (Figure 2S). Metformin treatment increased the protein levels of NICD and HES1 (Figure 2T,U). We then treated the cells with NOTCH inhibitor RO4929097 (RO) [12], which downregulated both NICD and HES1 protein levels (Figure 2V,W). qPCR results indicated that metformin increased the mRNA levels of key genes involved in tendon differentiation, including *TNMD* (Tenomodulin), *COL1A1*, and *RUNX2* (Figure 2X). While key target genes of Notch signaling were significantly altered (Figure 2X). Specifically, the upregulation of *TNMD* and *RUNX2* induced by metformin was blocked after RO treatment (Figure 2Y). Additionally, RO treatment downregulated the protein levels of pAKT and PCK1, but not other proteins involved in glucose and lipid metabolic pathways (Figure S11A,B). These data demonstrate that metformin administration protects against tendon damage by activating Notch signaling.

Strict control of T2DM has been proposed to decelerate the progression of diabetic tendinopathy and promote recovery. This includes administering glucose‐lowering drugs and dietary control for diabetic patients. Additionally, aerobic training, stretching, and strengthening exercises can prevent and alleviate tissue stiffness. However, the high risk of tendon injury in diabetic patients makes exercise challenging and heterogeneous, requiring personalized adjustments to avoid certain activities, such as intense and prolonged exercises. Additionally, medications and treatments, such as insulin, platelet‐rich plasma, aprotinin, corticosteroids, and anti‐inflammatory drugs, have shown some benefits in tendon healing in T2DM patients, but clinical evidence supporting their use in treating diabetic tendinopathy is limited [13]. In the present study, we showed that diabetic tendons lose matrix integrity and alter ECM and cell adhesion‐related gene expression. These results are generally consistent with previous findings, which show that pathological tendons lose matrix integrity and exhibit increased production of proteoglycans and glycosaminoglycans [14]. While T2DM patients taking metformin exhibited improved tendon structure and reduced levels of the tendon damage marker MMP3 [15], thus reducing the overall risk of severe tendon damage and surgery. Our data suggest that metformin acts as a direct regulator of tenogenic differentiation rather than through its anti‐hyperglycemic effects.

Diabetic tendinopathy is also characterized by impaired tenogenic differentiation. Studies have shown that hyperglycemia inhibits the proliferation and tenogenic differentiation ability of TSPCs, with decreased expression levels of the Scx, Col1a1, and TNMD [16]. We found that metformin directly activates the notch signaling during tenogenic differentiation and upregulates *TNMD*, *COL1A1*, and *RUNX2*, and the effects were specifically blocked by a Notch inhibitor RO4929097. Notch signaling is an evolutionarily conserved pathway that plays crucial functions in organ development, tissue homeostasis, stem cell fate choice, and metabolism [17]. The primary Notch target gene *Hes1* is found to regulate the expression of *Sox9* and *Runx2* during muscle cell differentiation, and inhibits osteoblast differentiation of BMSCs by inhibiting RUNX2 [18]. In addition, *HES1* expression marks explicitly a group of mesenchymal cells with chondrogenic capacity [19, 20]. It was possible that metformin promoted tenogenic differentiation and determined a specific lineage of tenocytes. However, whether and how notch signaling regulates tenogenic differentiation remains unclear, though we have applied multiple models to establish the connection between tendon damage, metformin, and notch signaling; more direct approaches, such as in vivo knockout mouse model and single‐cell sequencing would be ideal to further unravel the mechanism. In addition, a comparison of the effects of metformin with other antidiabetic agents, such as GLP‐1 receptor agonist, on diabetic tendinopathy, is also warranted in future studies.

In conclusion, we discovered that T2DM resulted in significant deltoid tendon damage and a higher risk of tendon surgery, attributed to abnormal tendon structure and impaired ECM and cell adhesion. Meanwhile, T2DM patients treated with metformin for hyperglycemia control had a reduced risk of tendon surgery, along with the downregulation of the key tendon damage‐related gene *MMP3* and the upregulation of the Notch signaling gene *HES1*. Additionally, cell culture, T2DM mice, and aged monkey models were also applied to confirm that metformin treatments upregulated Notch signaling in tendons. These results indicate that metformin directly protects tendons from injury by activating Notch signaling. Due to the difficulties in tendon sampling and processing, the current study only analyzed 77 human samples, which were smaller than expected to generate a stronger data set.

All the materials and methods are described in the Supporting Information.

## AUTHOR CONTRIBUTIONS

**Can Liu**: Conceptualization; methodology; investigation; funding acquisition; writing—original draft. **Runqi Wang**: Investigation; validation; formal analysis. **Yong Xu**: investigation; writing—review and editing; validation; formal analysis. **Qingyi Liu**: Investigation. **Yin Li**: Investigation. **Xiangpeng Liu**: Investigation. **Hui Shu**: Investigation. **Kaige Gao**: Investigation. **Xingye Zheng**: Investigation. **Yingying Dong**: Funding acquisition; Resources. **Yijun Wang**: Resources. **Bin Guo**: Resources. **Lei Fu**: Resources. **Bin Zhang**: Funding acquisition; resources; writing—review and editing. **Liang Zhao**: investigation; methodology; supervision; resources; writing—review and editing. **Zhihao Jia**: Conceptualization; writing—original draft; funding acquisition; writing—review and editing; supervision. **Xiaobo Sun**: Conceptualization; writing—review and editing; funding acquisition; supervision.

## CONFLICT OF INTEREST STATEMENT

The authors declare no conflicts of interest.

## ETHICS STATEMENT

The ethics application for mouse study (ZJ‐2021‐1) was approved by the CAM‐SU Animal Care and Use Committee. The ethics application for human deltoid tendons (2023‐lunli‐120) was approved by the Clinical Trial Ethics Committee of the Third Affiliated Hospital of Southern Medical University.

## Supporting information


**Figure S1:** Deltoid tendon biopsy collection and grouping profiling.
**Figure S2:** Quantification of DEGs from human deltoid tendon sequencing by different comparisons.
**Figure S3:** GO analysis of DEGs from different comparisons.
**Figure S4:** T2DM and obesity lead to impaired gene expression in ECM and immune‐related pathways in the deltoid tendon.
**Figure S5:** Female‐specific comparative analyses between Control and DB groups.
**Figure S6:** Specific comparative analyses between DB and DB+Met groups.
**Figure S7:** GO enrichment analysis of DEGs identified in comparisons between the DB group and DB+Met group.
**Figure S8:** Metformin suppresses *MMP3* but activates *HES1* in aged monkey tendon.
**Figure S9:** Metformin improved diabetic symptoms in DB/DB mice.
**Figure S10:** GO enrichment analysis of DEGs in deltoid tendon tissues from the DB group versus DB+Met group.
**Figure S11:** RO selectively downregulates pAKT and PCK1 in BMSC‐derived tenocytes.

**Table S1:** Gene Expression profiling of human deltoid tendon.
**Table S2:** CONTROL vs DB from human deltoid tendon.
**Table S3:** CONTROL vs OB from human deltoid tendon.
**Table S4:** OW vs OB from human deltoid tendon.
**Table S5:** CONTROL vs OW from human deltoid tendon.
**Table S6:** DB vs DB‐Met from human deltoid tendon.
**Table S7:** Gene Expression profiling of mouse deltoid tendon from WT, WT+Met, DB and DB+Met groups.
**Table S8:** DB vs WT from mouse deltoid tendon.
**Table S9:** DB+Met vs DB from mouse deltoid tendon.
**Table S10:** WT vs WT+Met from mouse deltoid tendon.

## Data Availability

The data that support the findings of this study are available on request from the corresponding author. The data are not publicly available due to privacy or ethical restrictions. All data and materials used in the analyses were included in the main text and supplementary. The data and scripts used are saved in GitHub https://github.com/KNDR10/imeta. Supplementary materials (methods, figures, tables, graphical abstract, slides, videos, Chinese translated version, and update materials) may be found in the online DOI or iMeta Science http://www.imeta.science/.
