# Supplementary material for: Metformin Administration Protects Against Deltoid Tendon Damage Through Activation of Notch Signaling
Source: Imeta. 2025 Aug 31;4(5):e70074. doi: 10.1002/imt2.70074 (PMC12528006; doi:10.1002/imt2.70074)
Supplement: Supplementary file 2 — Table S1: Gene Expression profiling of human deltoid tendon. Table S2: CONTROL vs DB from human deltoid tendon. Table S3: CONTROL vs OB from human deltoid tendon. Table S4: OW vs OB from human deltoid tendon. Table S5: CONTROL vs OW from human deltoid tendon. Table S6: DB vs DB‐Met from human deltoid tendon. Table S7: Gene Expression profiling of mouse deltoid tendon from WT, WT+Met, DB and DB+Met groups. Table S8: DB vs WT from mouse deltoid tendon. Table S9: DB+Met vs DB from mouse deltoid tendon. Table S10: WT vs WT+Met from mouse deltoid tendon. [file IMT2-4-e70074-s001.docx]

**Supporting information to:**

**Metformin administration protects deltoid tendon damage through activation of Notch signaling**

Can Liu^1, 2, #^, Runqi Wang^3, #^, Yong Xu^4, #^, Qingyi Liu^3^, Yin Li^3^, Xiangpeng Liu^3^, Hui Shu^3^, Kaige Gao^3^, Xingye Zheng^3^, Yingying Dong^3^, Yijun Wang^1, 2^, Bin Guo^5^, Lei Fu^5^, Bin Zhang^6,^ *, Liang Zhao^1, 2,^ *, Zhihao Jia^3,^ *, Xiaobo Sun^7,^ ^8, 9^ *

^1^Department of shoulder and elbow surgery, Center for Orthopedic Surgery, The Third Affiliated Hospital of Southern Medical University, Guangzhou 510630, China

^2^Shoulder Research Institute, Academy of Orthopedics, Guangzhou 510630, China

^3^Cambridge-Suda Genomic Resource Center, Suzhou Medical College, Soochow University, Suzhou 215123, China.

^4^Orthopaedic Institute, Suzhou Medical College, Soochow University, Suzhou 215123, China

^5^Wisdom Lake Academy of Pharmacy, Xi'an Jiaotong-Liverpool University, Suzhou 215123, China.

^6^Institute of Medicinal Plant Development, Peking Union Medical College and Chinese Academy of Medical Sciences, Beijing 100193, China

^7^Collaborative Innovation Center of Prevention and Treatment of Major Diseases by Chinese and Western Medicine, Henan Province, China

^8^Henan University of Chinese Medicine, Zhengzhou 450046, China

^9^The First Affiliated Hospital of Henan University of Chinese Medicine, Zhengzhou 450046, China

^#^These authors contributed equally: Can Liu, Runqi Wang, Yong Xu

*Correspondence: zhangbin7@126.com (Bin Zhang) [zhaoliang717@sina.com](mailto:zhaoliang717@sina.com) (Zhao liang) [zhjia@suda.edu.cn](mailto:zhjia@suda.edu.cn) (Zhihao Jia) [sun_xiaobo163@163.com](mailto:sun_xiaobo163@163.com) (Xiaobo Sun)

Bin Zhang, Institute of Medicinal Plant Development, Peking Union Medical College and Chinese Academy of Medical Sciences, Beijing 100193, China. Email: zhangbin7@126.com

Zhao liang, Department of shoulder and elbow surgery, Center for Orthopedic Surgery, The Third Affiliated Hospital of Southern Medical University, Guangzhou 510630, China; Shoulder Research Institute, Academy of Orthopedics, Guangdong Province, China. Email: [zhaoliang717@sina.com](mailto:zhaoliang717@sina.com)

Zhihao Jia, Cambridge-Suda Genomic Resource Center, Suzhou Medical College, Soochow University, Suzhou 215123, China. Email: [zhjia@suda.edu.cn](mailto:zhjia@suda.edu.cn)

Xiaobo Sun, Academy of Chinese Medical Sciences, Henan University of Chinese Medicine, Zhengzhou 450046, China. Email: [sun_xiaobo163@163.com](mailto:sun_xiaobo163@163.com)

**METHODS**

**Animal care**

Wild-type (WT) mice used in this study were in a C57BL/6 backgroud, which were bred and maintained in a specific pathogen-free (SPF) facility at CAM-SU (Suzhou, China). DB/DB mice were purchased from Cyagen Biosciences. The mice were provided with unrestricted access to acidified water and standard irradiated and autoclaved rodent diet, and were kept on an 8 a.m. to 8 p.m. light-dark cycle. Animal care and experimental procedures adhered to the approved protocol ZJ-2021-1, as sanctioned by the Institutional Animal Care and Use Committee (IACUC) of CAM-SU on December 24, 2021.

**Human tendon sample collection**

Human deltoid tendons were collected from Department of shoulder and elbow surgery, Center for Orthopedic Surgery, The Third Affiliated Hospital of Southern Medical University during tendon surgery under the protocol 2023-lunli-120 approved by Clinical Trial Ethics Committee of the Third Affiliated Hospital of Southern Medical University on December 27, 2023.

**Metformin treatment of diabetic mice**

DB/DB mice were purchased from Cyagen Biosciences and 8-week-old male DB/DB mice were subjected oral metformin administration for 2 months. Metformin was given in drinking water with a dose of 200mg/kg/day and the water was replaced for every two days.

For streptozotocin (STZ)-induced T2DM mouse model, 8-week-old male WT C57BL/6 mice were first feed with a high-fat diet (HFD) for 4 weeks. Then received a single intraperitoneal injection of STZ (80 mg/kg, Sigma-Aldrich, St. Louis, MO). STZ was freshly prepared by dissolving in citrate buffer. After one week of injection, success diabetic mice were confirmed by measurements of fasting glucose concentrations >11.1 mmol/L. Then the T2DM mice were subjected to oral metformin administration for 8 weeks. Metformin was given as described above.

**Establishment of a Tendon Injury Model in Mice**

Eight-week-old wild-type mice were chosen to undergo tendon injury induction through treadmill exercise. The mice participated in treadmill training five days per week, with rest on weekends, for eight weeks. Each session lasted for one hour. During the initial 30 minutes, the speed was set at 10 m/min, while the inclination was incrementally increased by 1 degree per minute, reaching a peak of 30 degrees. In the following 30 minutes, the incline was maintained at 30 degrees, and the speed gradually rose to 15 m/min over the first 10 minutes. The final 20 minutes of each session involved a constant speed of 15 m/min at an inclined 30 degrees.

The Met group was subjected to treadmill exercise and administered subcutaneous metformin injections at a dosage of 25 mg/kg. The Untreated group did not engage in treadmill exercise but received subcutaneous injections of an equivalent volume of saline, administered concurrently with the Met group. The Control group participated in treadmill exercise and was given subcutaneous injections of an equivalent volume of saline, mirroring the treatment protocol of the Met group. All three groups received their respective treatments for two weeks prior to the initiation of treadmill training.

**The treadmill endurance test**

The treadmill speed was maintained at a constant rate of 10 m/min. The incline gradually increased from 0° to 30° over the course of 45 minutes, with a 2° increase every 3 minutes. Exhaustion was defined as the point when the total number of shocks reached 250 or the duration of individual shocks exceeded 10 seconds.

**Grip Strength test**

Grip strength was assessed for both the forelimbs and all four limbs of the mice. For forelimb grip strength measurement, the mouse was placed on the grip strength meter with its hind paws suspended, allowing only the forepaws to grasp the apparatus. To measure the grip strength of all four limbs, the mouse was positioned to grasp the apparatus with all four limbs simultaneously, while the tail was gently pulled similarly. Each test was conducted three times, and the average value was recorded.

**Western Blots**

Tendon tissue obtained from dissection is stored in liquid nitrogen. Once removed, 150 µL of RIPA lysis buffer, containing protease and phosphatase inhibitors, is added to each EP tube. The tendon tissue is homogenized using a handheld homogenizer, followed by ultrasonic treatment to ensure complete tissue disruption. The EP tubes are then incubated at 4°C for 1 hour, then centrifuged to collect the supernatant for BCA protein quantification. For electrophoresis, the samples are first run at 80V through the stacking gel, with the voltage subsequently increased to 120V until the process is complete. Equal proteins are then transferred onto a polyvinylidene fluoride (PVDF) membrane (Millipore Corporation). After blocking with non-fat milk for 1 hour, the membrane is incubated overnight with the primary antibody at 4°C.

The primary antibodies employed in this study included Anti-HES1 (BM4488, BOSTER), Anti-ACTIN (66009-1-Ig, Proteintech), Anti-NICD (20687-1-AP, Proteintech), Anti-Tubulin (2146S, CST), Anti-GAPDH (60004-1-Ig, Proteintech), Anti-Phospho-Akt (9271S, CST), Anti-PCK1 (16754-1-AP, Proteintech), Anti-PDHK1 (3820, CST), Anti-PGK1 (68540, CST), Anti-PKM1/2 (3190, CST), Anti-LDHA (2012, CST), Anti-CPT2 (52552, CST), Anti-ATGL (M01800, BOSTER), and Anti-HSL (4107, CST).

The next day, the PVDF membrane with bound proteins is incubated with the secondary antibody (Jackson ImmunoResearch) in a milk solution at room temperature for 1 hour. Signal detection was carried out using an enhanced chemiluminescence (ECL) substrate (Vazyme), and the resulting signals were captured using a chemiluminescence imaging system (ChemiScope 6100 Touch).

**Section and H&E Staining**

Tissue samples fixed in formalin were washed with 0.9% saline. Decalcification was carried out using an EDTA solution. Following decalcification, the samples were embedded in an Optimal Cutting Temperature (OCT) compound and frozen in liquid nitrogen. The following day, the embedded tissue was sectioned to a thickness of 10 μm. The sections were then placed in a 37 °C oven for fixation. Once prepared, the sections could be stored in a -20 °C freezer.

The slides were first immersed in hematoxylin for 40 seconds, then rinsed with distilled deionized water until the wash water became clear. The sections were sequentially rinsed in 50%, 75%, and 95% ethanol, each lasting one minute. After staining with eosin for 7 seconds, the sections were rinsed twice with 95% ethanol, each rinse lasting one minute. Subsequently, the sections were washed twice with anhydrous ethanol for five minutes per rinse, followed by two washes with xylene, each lasting five minutes. Finally, the sections were mounted with neutral balsam.

**Total RNA Extraction and Real-time PCR**

According to the manufacturer's protocol, total RNA was isolated from cells or tissues using TRIzol reagent (Life Technologies). RNA purity and concentration were assessed with a spectrophotometer (NanoDrop 2000, Thermo Fisher Scientific), measuring 260 nm and 280 nm absorbance. All samples exhibited 260/280 nm absorbance ratios near 2, indicating high purity.

RNA was then reverse transcribed into cDNA using random primers and M-MLV reverse transcriptase. Real-time PCR was performed on a Roche LightCycler 480 PCR System, utilizing SYBR Green Master Mix (Vazyme) and gene-specific primers obtained from PrimerBank or designed from sequences in the NCBI database. Relative gene expression changes were calculated using the 2−ΔΔCT method, with β-Actin as the internal control.

**Calorimetric Assessment**

An indirect calorimetry system (Oxymax, Columbus Instruments, Columbus, OH, USA) was employed to measure oxygen consumption (VO₂) and carbon dioxide production (VCO₂) in mice during both day and night periods. The system was located in the animal facility at CAM-SU, where environmental conditions were rigorously controlled: the temperature was set at 24 °C, and the light-dark cycle was maintained with 12 hours of light (8:00 a.m.–8:00 p.m.) and 12 hours of darkness (8:00 p.m.–8:00 a.m.). Each mouse was placed in a chamber with unrestricted access to food and water. The experiment lasted for three days, with the first day designated for acclimation to the chambers. Data were normalized to body weight to account for energy expenditure. Average energy expenditure during the day (8:00 a.m.–8:00 p.m.) and night (8:00 p.m.–8:00 a.m.) was calculated by averaging all recorded data points within these time intervals.

**Bone marrow-derived stem cells (BMSC) culture and differentiation**

The freshly revived BMSC were initially cultured in 10 cm dishes. Once the cell density reached approximately 70%, the cells were transferred to a six-well plate for experimental grouping. Each well contained 2 mL of BMSC-specific culture medium. In the metformin treatment experiment involving undifferentiated BMSC, the cells were divided into Control, Met (10 μM), and Met (10 nM). The control group was treated with 0.1% DMSO. After one day of treatment, samples were collected for further analysis.

Differentiation of BMSC was induced when the cell density reached 85-95%. After washing each well with PBS, a serum-free BMSC-specific culture medium was added to starve the cells, facilitating their transition into the differentiation state. After one day, the cells were removed from the incubator, washed with PBS, and cultured in a differentiation medium containing BMP-12 to initiate differentiation. After another day, the medium was replaced with a regular culture medium. The cells were maintained for 13 days, with the medium changed every two days. The groups were divided into Control, Met (10 nM), and Met + RO4929097 inhibitor (10 μM). After one day of treatment, samples were collected for further analysis.

**mRNA library constructing and sequencing**

1μg total RNA was used for following library preparation. The poly(A) mRNA isolation was performed using Oligo(dT) beads. The mRNA fragmentation was performed using divalent cations and high temperature. Priming was performed using Random Primers. First strand cDNA and the second-strand cDNA were synthesized. The purified double-stranded cDNA was then treated to repair both ends and add a dA-tailing in one reaction, followed by a T-A ligation to add adaptors to both ends. Size selection of Adaptor-ligated DNA was then performed using DNA Clean Beads. Each sample was then amplified by PCR using P5 and P7 primers and the PCR products were validated.

Then libraries with different indexs were multiplexed and loaded on an Illumina HiSeq/ Illumina Novaseq/ MGI2000 instrument for sequencing using a 2x150 paired-end (PE) configuration according to manufacturer’s instructions.

**Expression analysis**

In the beginning transcripts in fasta format are converted from known gff annotation file and indexed properly. Then, with the file as a reference gene file, HTSeq (v0.6.1) estimated gene and isoform expression levels from the pair-end clean data.

**Differential expression analysis**

Differential expression analysis used the DESeq2 Bioconductor package, a model based on the negative binomial distribution. the estimates of dispersion and logarithmic fold changes incorporate data-driven prior distributions, Padj of genes were setted <0.05 to detect differential expressed ones.

**GO and KEGG enrichment analysis**

GOSeq(v1.34.1) was used identifying GO terms that annotate a list of enriched genes with a significant padj less than 0.05. And topGO was used to plot DAG.

KEGG is a collection of databases dealing with genomes, biological pathways, diseases, drugs, and chemical substances (http://en.wikipedia.org/wiki/KEGG). We used scripts in house to enrich significant differential expression gene in KEGG pathways.

**Statistical Analysis**

Data was analyzed using GraphPad Prism 8.0 (GraphPad Software, CA, USA). All analyses between two groups were conducted with Student’s t test (two-sided), the correlation analysis of T2DM/metformin to surgery was conducted by Pearson Chi-square test, multiple comparisons of results were conducted by two-way ANOVA and ANCOVA analysis. All experimental data are presented as mean ± SEM (standard error of mean). Comparisons with two-sided p values < 0.05 were considered statistically significant.

**
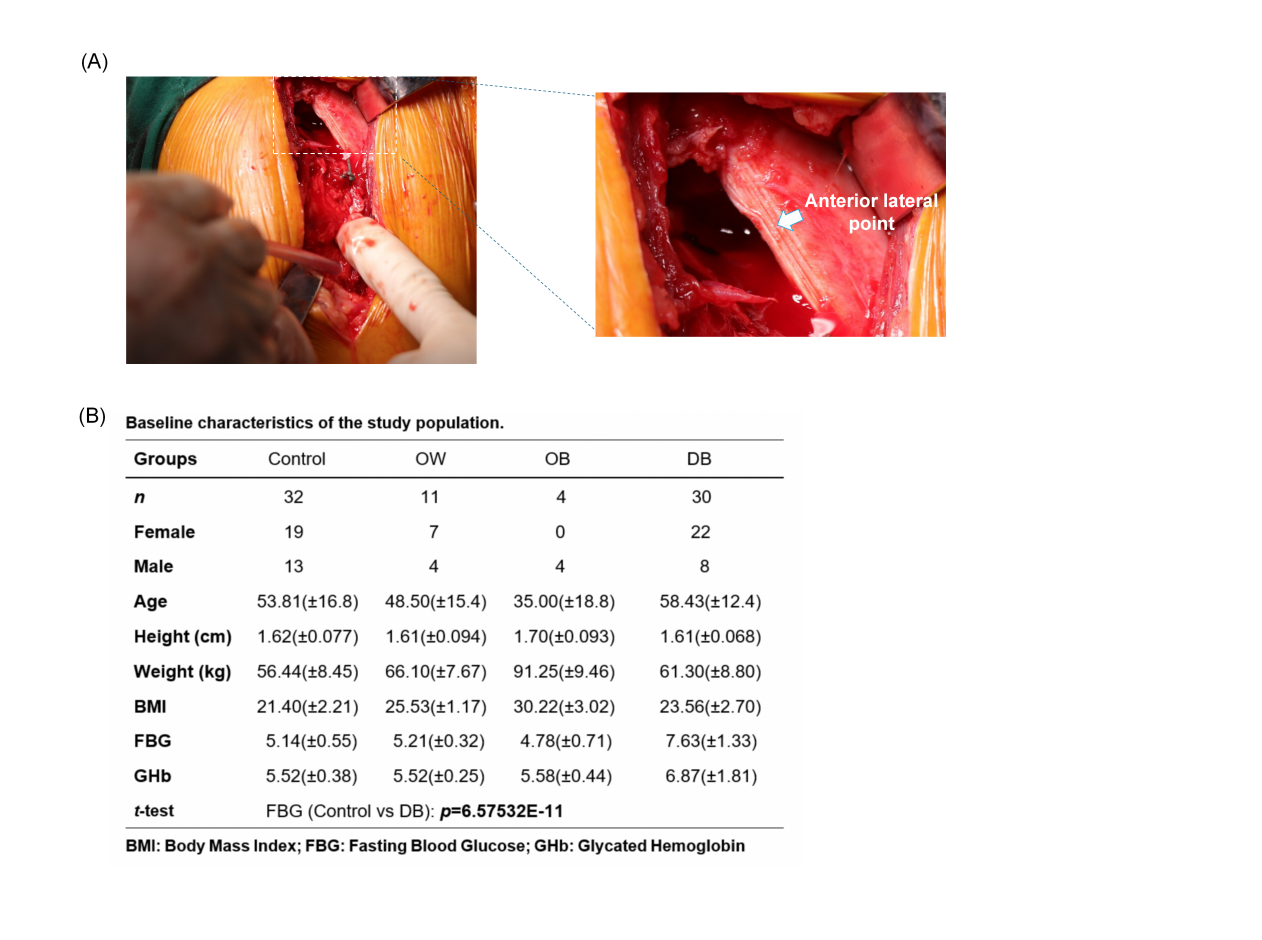
Figure S1** Deltoid tendon biopsy collection and grouping profiling. (A) Tendon samples were collected from the tendon fascicles inserting into the acromion. (B) Baseline characteristics of the study cohort, stratified into four groups according to BMI and diabetes diagnosis: Control, OW, OB, and DB. Data include sex, age, height, weight, BMI, FBG, and GHb.


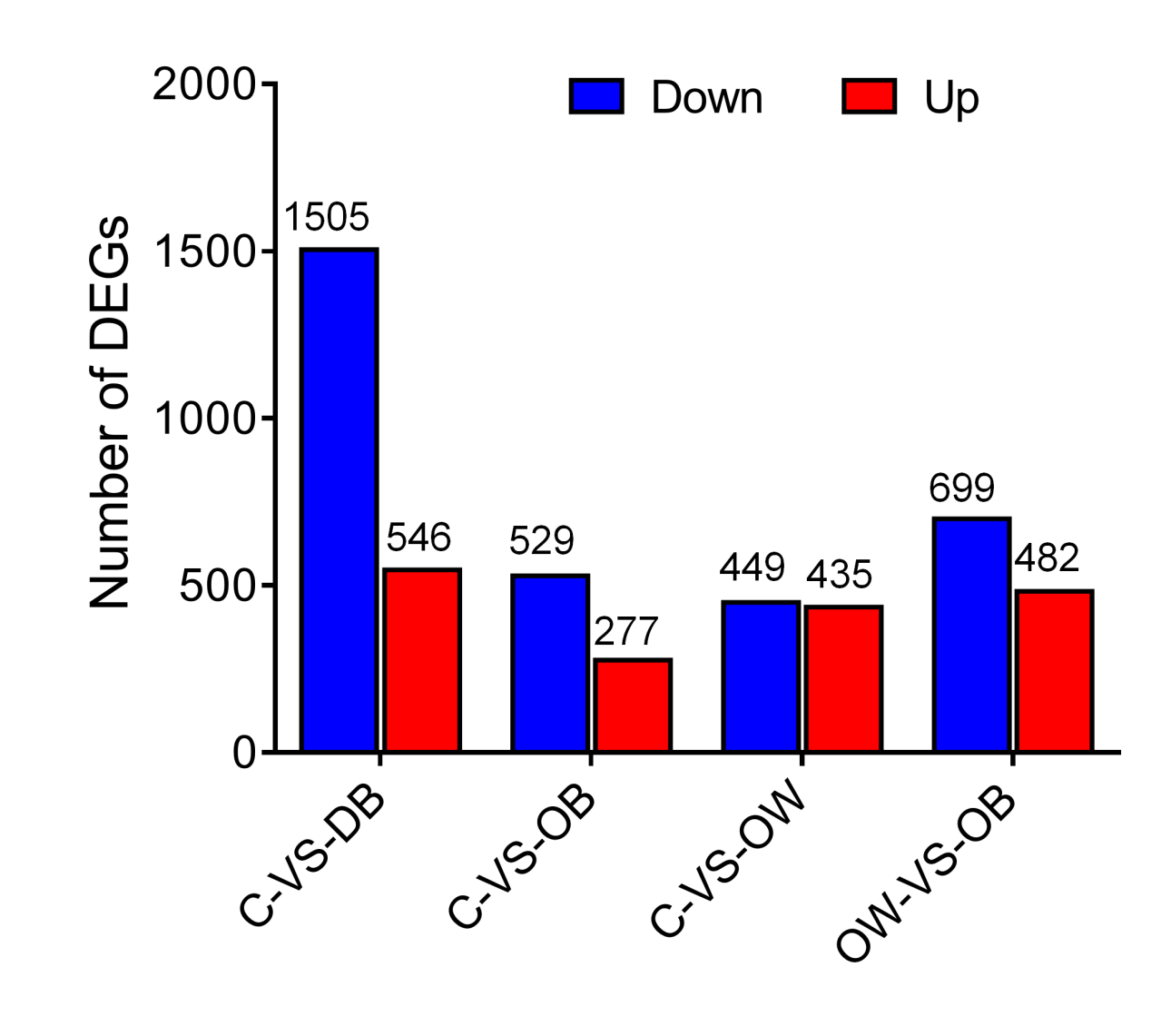


**Figure S2** Quantification of DEGs from human deltoid tendon sequencing by different comparisons. Comparative analysis of DEGs was performed between: Control and DB groups, Control and OB groups, Control and OW groups, OW and OB groups.


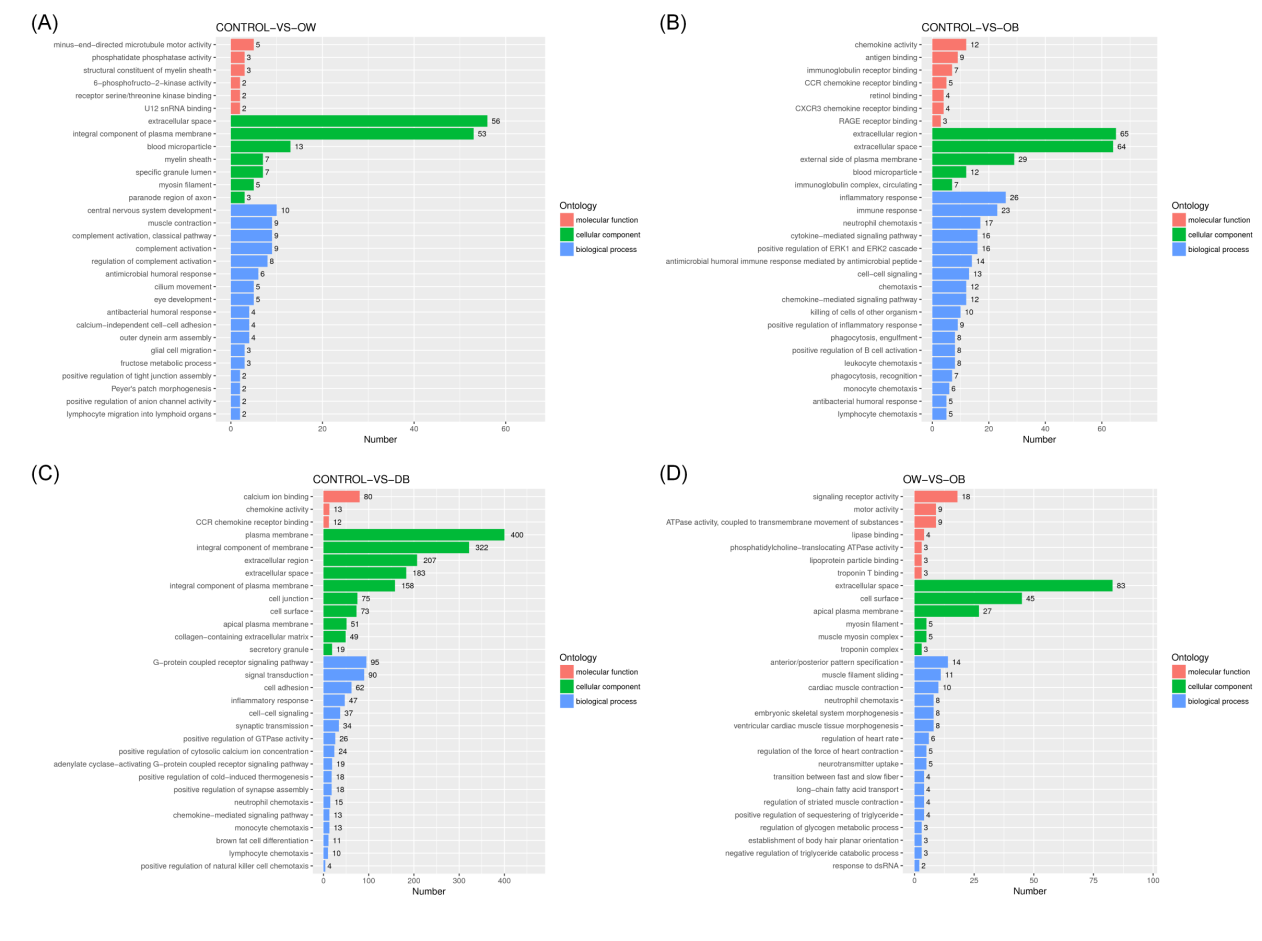


**Figure S3** GO analysis of DEGs from different comparisons. (A) GO analysis of comparisons between Control and OW groups. (B) GO analysis comparing Control and OB groups. (C) GO analysis for Control versus DB groups. (D) Comparative GO analysis of OW and OB groups.​


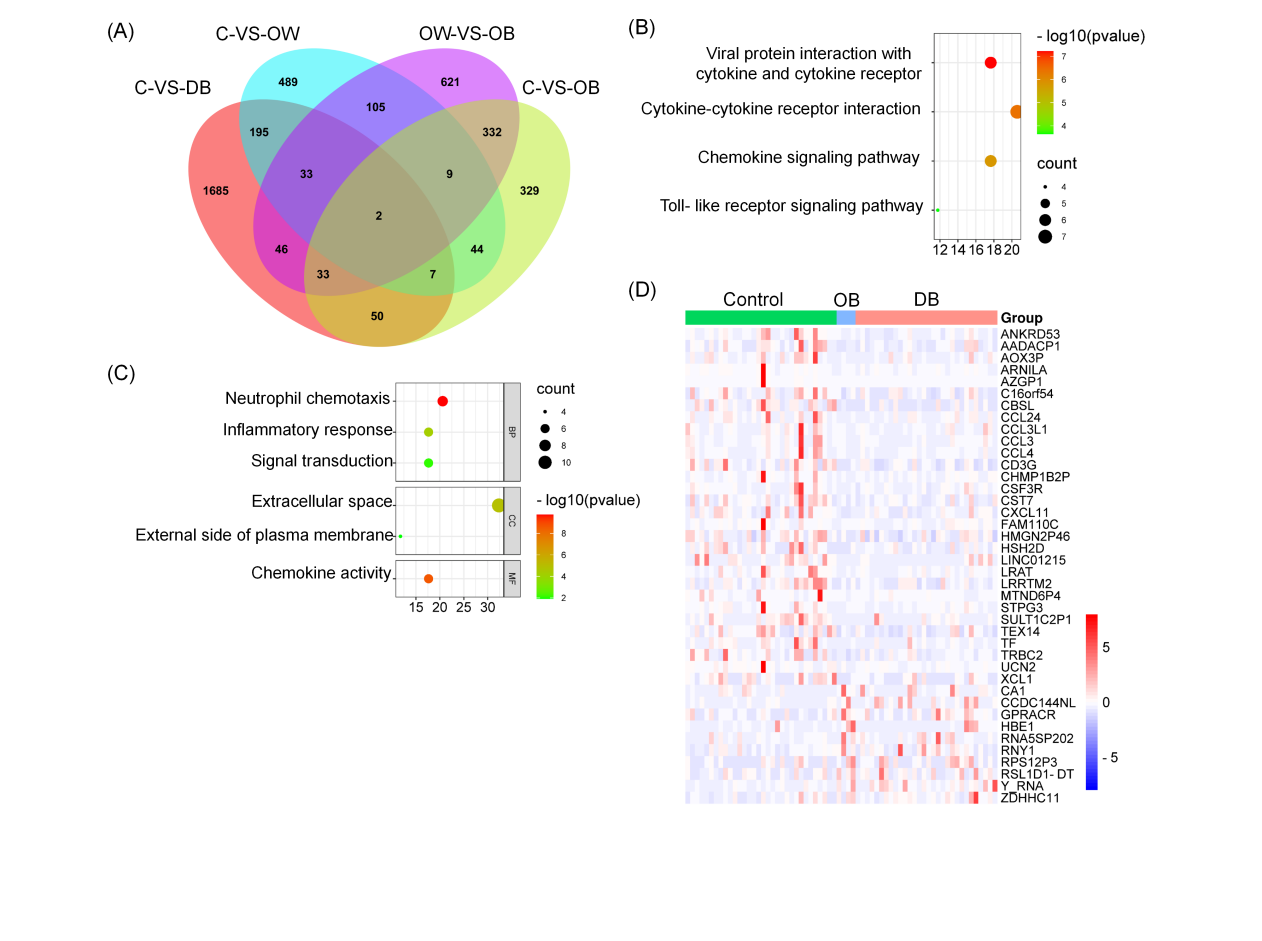


**Figure S4** T2DM and obesity lead to impaired gene expression in ECM and immune-related pathways in the deltoid tendon. (A) Overlap analysis of DEGs across all four groups. (B) KEGG functional analysis of overlapping DEGs. (C) GO analysis of overlapping DEGs. (D) Cluster heatmap analysis for Control, OB, and DB groups.


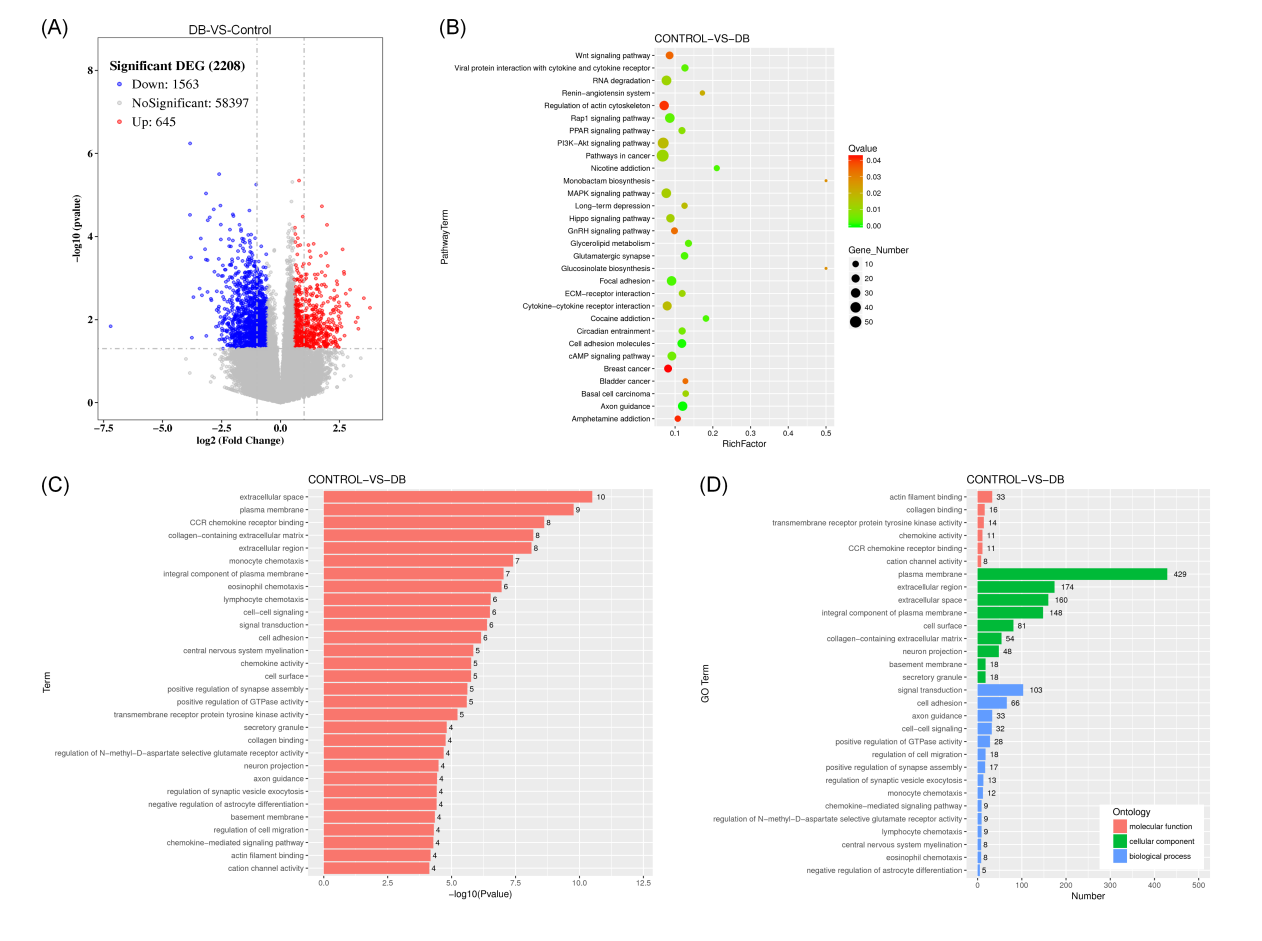


**Figure S5** Female-specific comparative analyses between Control and DB groups. (A-D) Female-specific comparative analyses between Control and DB groups were conducted for DEGs, GO enrichment, KEGG pathway annotation.


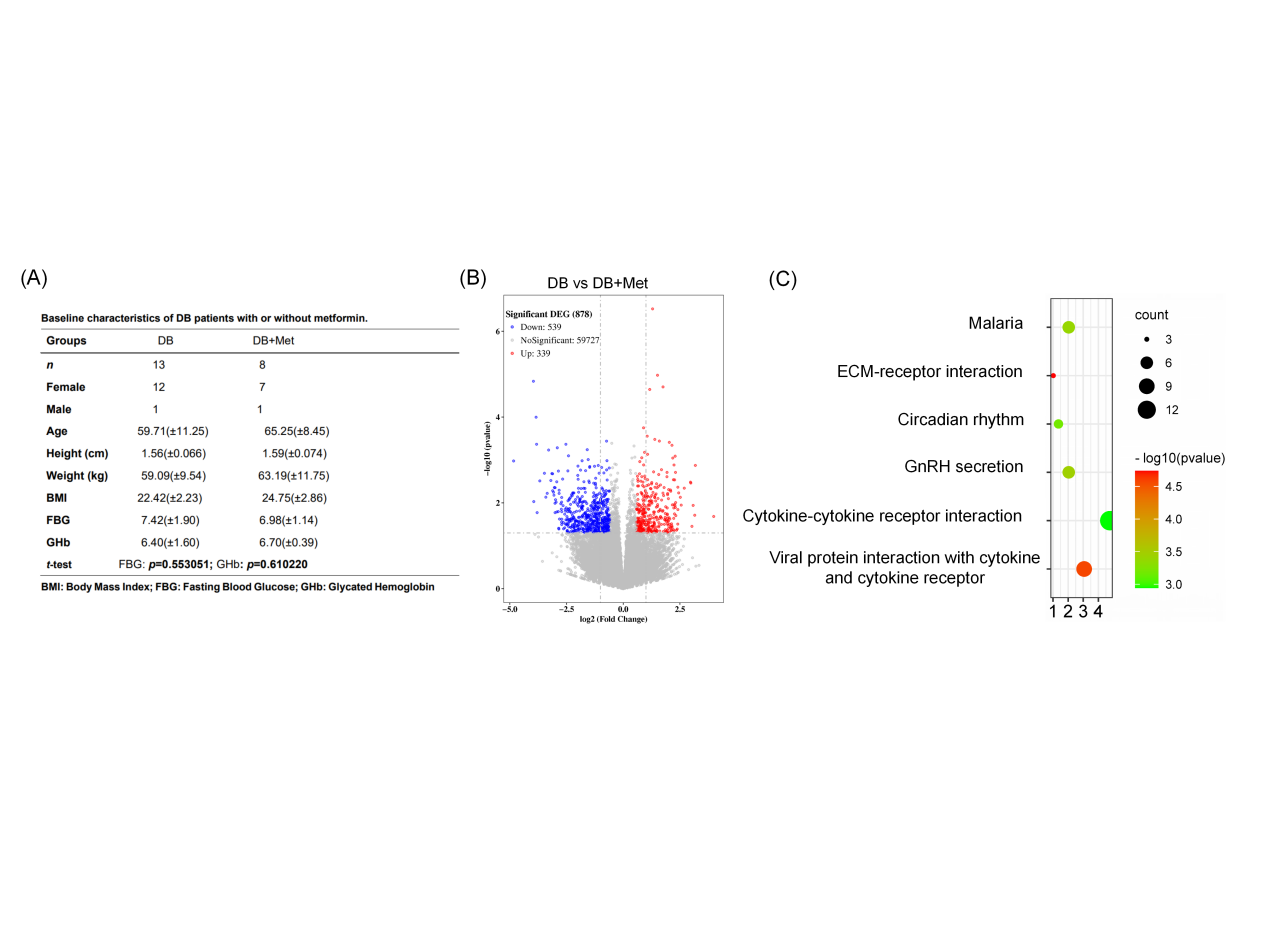


**Figure S6** Specific comparative analyses between DB and DB+Met groups. (A) Baseline characteristics of the DB group and metformin-treated DB group (DB+Met), including sex, age, height, weight, BMI, FBG, and GHb. (B) Volcano plot visualization of DEGs between DB and DB+Met groups. (C) KEGG pathway enrichment analysis of DEGs identified in DB versus DB+Met comparisons.​​

**
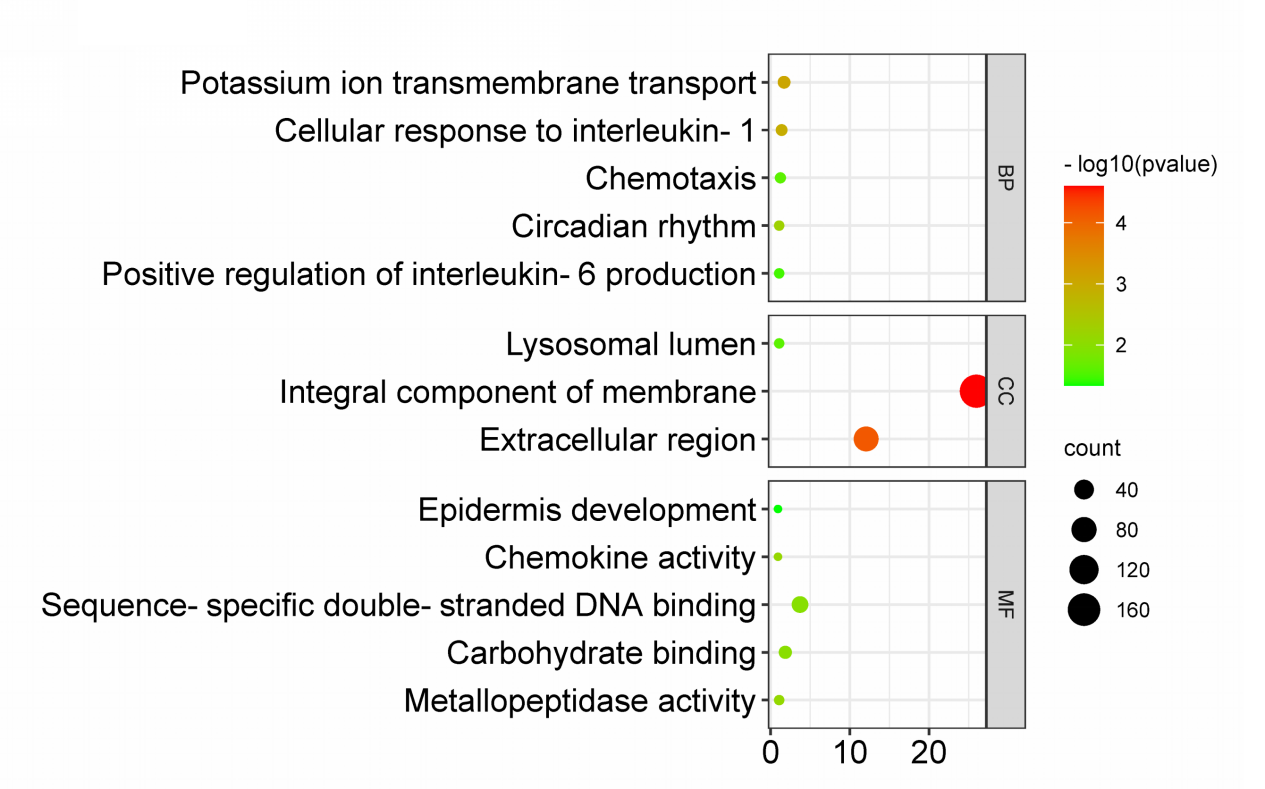
**

**Figure S7** GO enrichment analysis of DEGs identified in comparisons between the DB group and DB+Met group. GO enrichment analysis of DEGs identified in comparisons between the DB group and DB+Met group.​


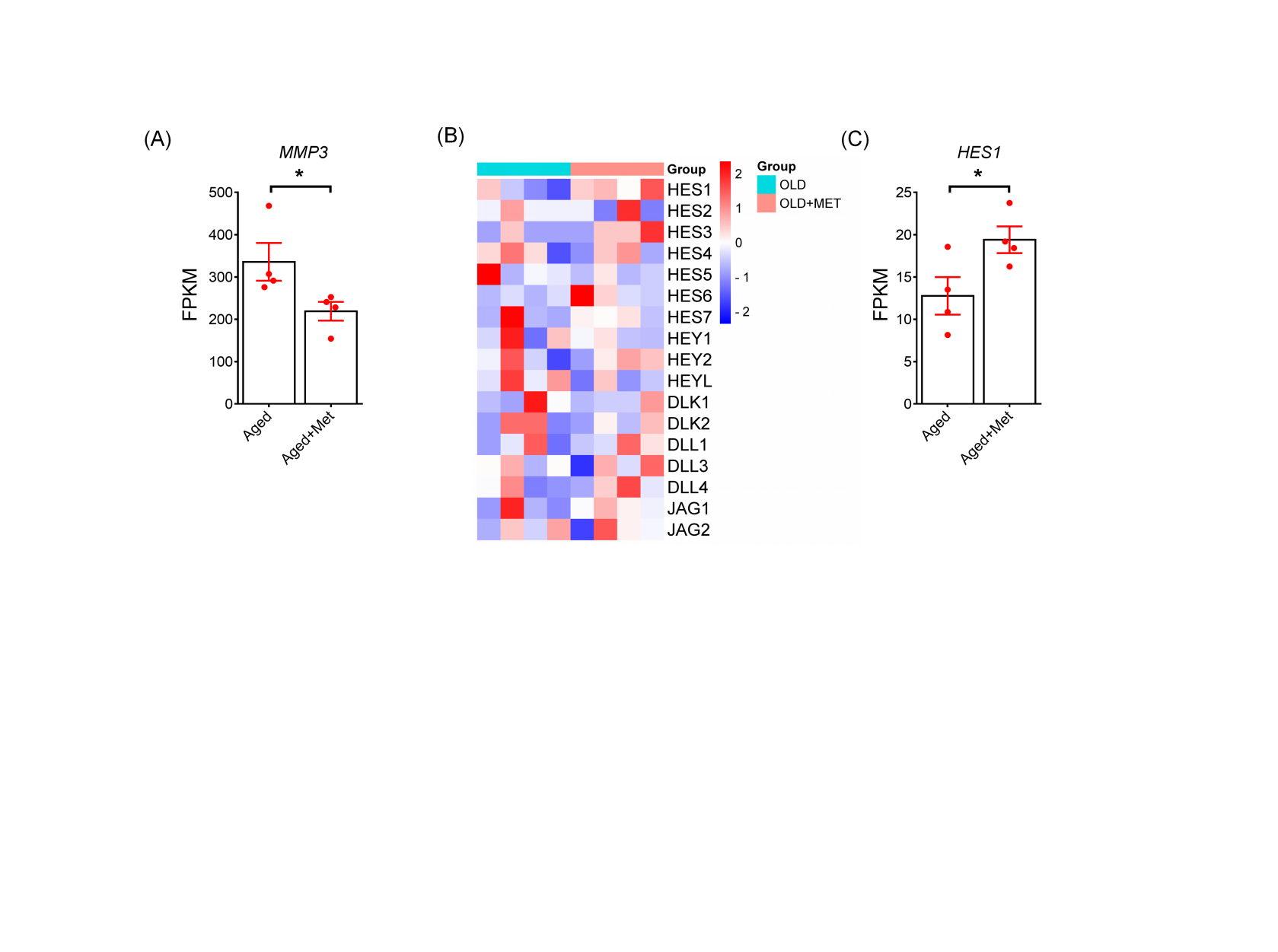


**Figure S8** Metformin suppresses *MMP3* but activates *HES1* in aged monkey tendon. (A) RNA-seq quantification of MMP3 expression in tendon tissues from aged monkey Control and Metformin-treated groups. (B) Hierarchical clustering heatmap analysis of tendon RNA-seq profiles comparing aged monkey Control versus Metformin cohorts. (C) HES1 transcript abundance measured via RNA-seq in tendon samples of aged monkeys under Control and Metformin interventions. Data represent mean ± SEM (t-test: **p* < 0.05). The RNA-seq data from aged monkeys were derived from the source cited in the online version of this article via DOI: <https://doi.org/10.1016/j.cell.2024.08.021>.


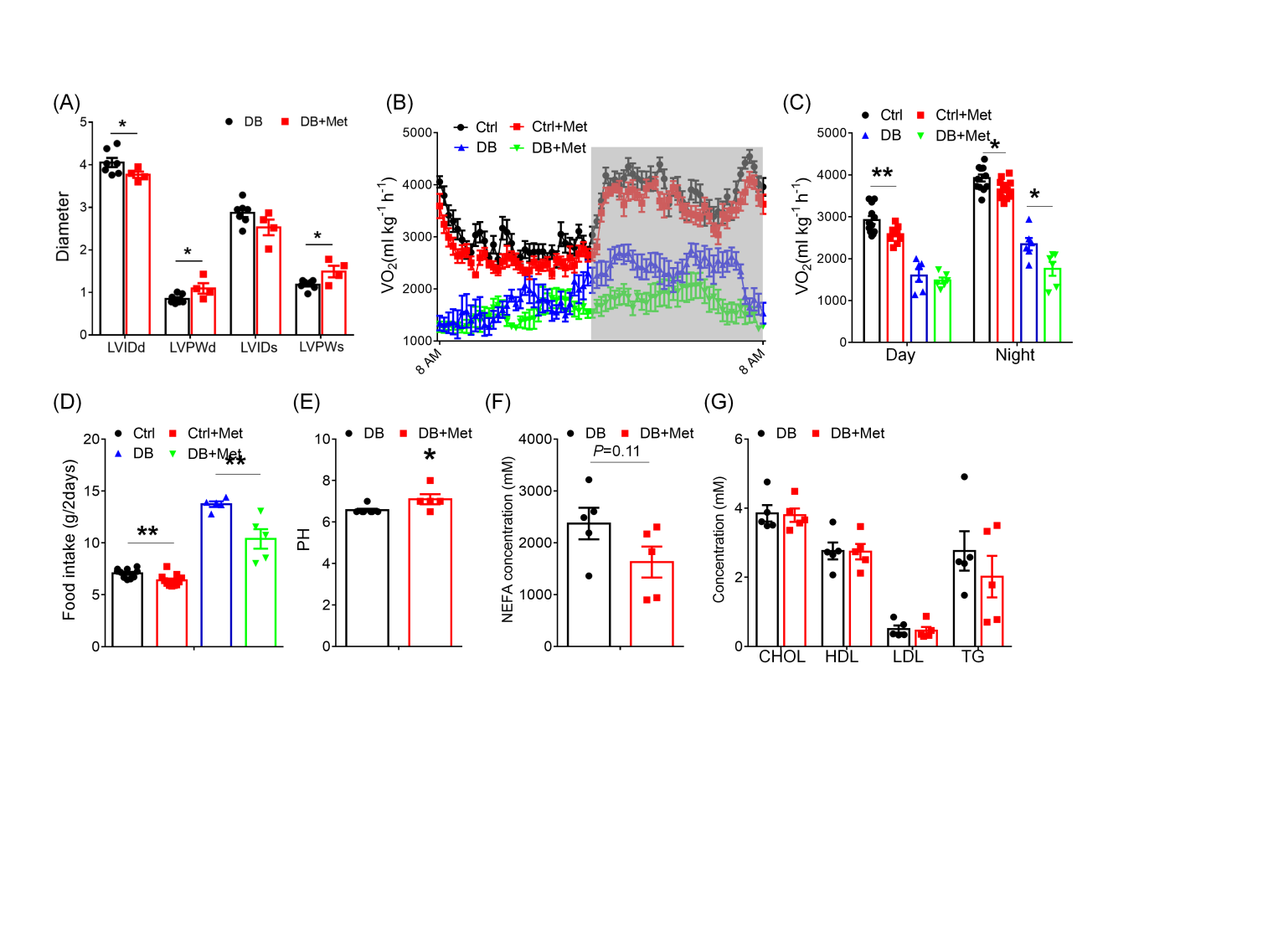


**Figure S9** Metformin improved diabetic symptoms in DB/DB mice. (A) Echocardiographic parameters in DB/DB mouse model: Control vs. metformin-treated (Met) groups. Data represent left ventricular internal diameter in diastole (LVIDd), posterior wall thickness in diastole (LVPWd), left ventricular internal diameter in systole (LVIDs), and posterior wall thickness in systole (LVPWs). (B, C) Oxygen consumption (VO₂) during light and dark phases in Control and Met-treated DB/DB mice. (D) Daily food intake measurements in Control and Met cohorts of DB/DB mice. (E) Urinary pH values from spot urine samples in Control versus Met-treated DB/DB groups. (F, G) Plasma lipid profiles of Control and Met DB/DB groups, including triglycerides and total cholesterol. Data represent mean ± SEM (t-test, two-way ANOVA and ANCOVA: **p* < 0.05, ***p* < 0.01).


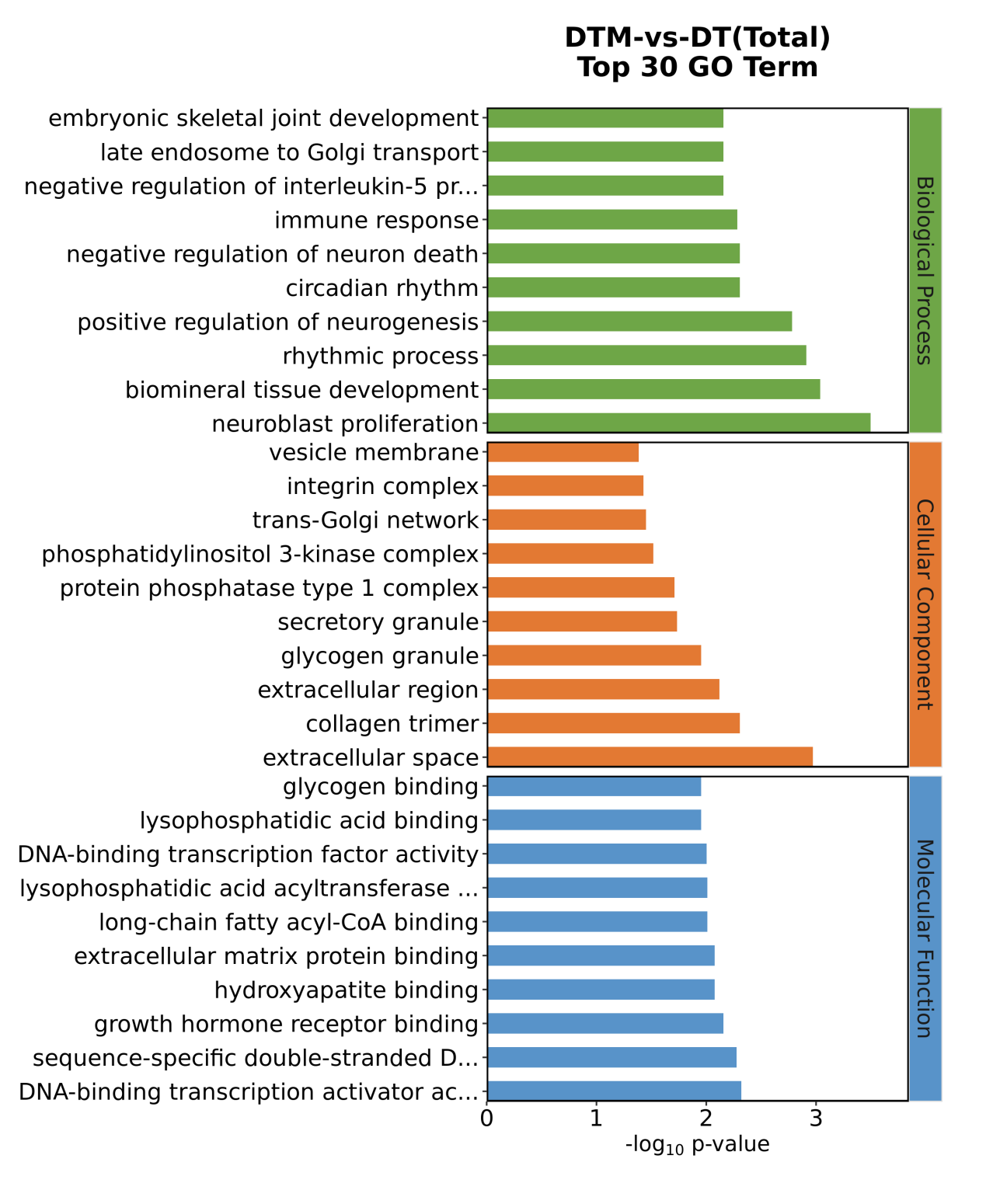


**Figure S10** GO enrichment analysis of DEGs in deltoid tendon tissues from the DB group versus DB+Met group. GO enrichment analysis of DEGs in deltoid tendon tissues from the DB group versus DB+Met group.​


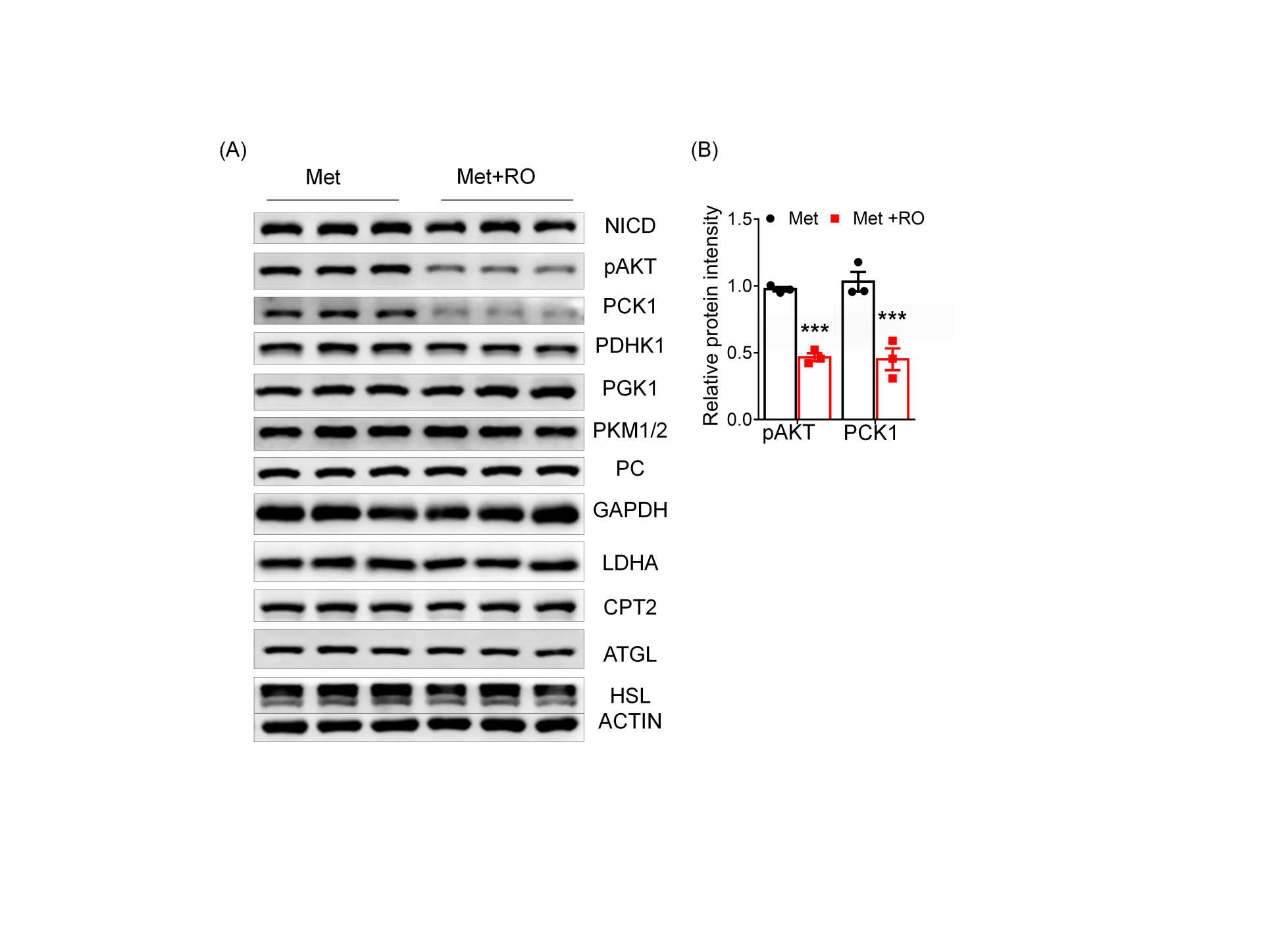


**Figure S11** RO selectively downregulates pAKT and PCK1 in BMSC-derived tenocytes. (A, B) WB of proteins in BMSC-derived tendon cells treated with Met vs Met+RO. Targets: NICD, pAKT, PCK1, PDHK1, PGK1, PKM1/2, PC, GAPDH, LDHA, CPT2, ATGL, HSL, ACTIN. Data represent mean ± SEM (t-test: ****p* < 0.001).
